# A comprehensive review of genomic-scale genetic engineering as a strategy to improve bacterial productivity

**DOI:** 10.1099/mic.0.001628

**Published:** 2025-11-03

**Authors:** Mario Alberto Pantoja-Alonso, José Alberto Camas-Reyes, Rafael Cano-Segura, María del Rosario Cárdenas-Aquino, Agustino Martínez-Antonio

**Affiliations:** Biological Engineering Laboratory, Cinvestav Irapuato, Km. 9.6 Libramiento Norte Carr, Irapuato-León 36824 Irapuato Gto, Mexico1

**Keywords:** CRISPR/Cas systems, genome editing, genomic tools and high-value metabolites, metabolic engineering, synthetic biology

## Abstract

Bacterial genome engineering has evolved to provide increasingly precise, robust and rapid tools, driving the development and optimization of bacterial production of numerous compounds. The field has progressed from early random mutagenesis methods, labour-intensive and inefficient, to rational and multiplexed strategies enabled by advances in genomics and synthetic biology. Among these tools, CRISPR/Cas has stood out for its versatility and its ability to achieve precision levels ranging from 50% to 90%, compared to the 10–40% obtained with earlier techniques, thereby enabling remarkable improvements in bacterial productivity. Nevertheless, like its predecessors, it still demands continuous refinement to reach full maturity. In this context, the present review addresses the lack of a unified overview by summarizing historical milestones and practical applications of genomic engineering tools in bacteria. It integrates diverse approaches to provide a comprehensive perspective on the evolution and prospects of these fundamental biotechnological tools.

## Introduction

Bacteria have been extensively employed in the production of metabolites, proteins, additives, fuels and other compounds of interest [1]. However, to meet commercial demands, it was necessary to develop bacterial cells capable of achieving high productivity, defined as the total concentration of a compound produced in a bacterial culture within a given time frame. In this context, metabolic engineering (ME), together with the emergence of rational genome engineering (RGE), largely driven by synthetic biology (SB), significantly expanded the capacity to design and generate bacteria with optimized productive traits [2].

**Fig. 1.**
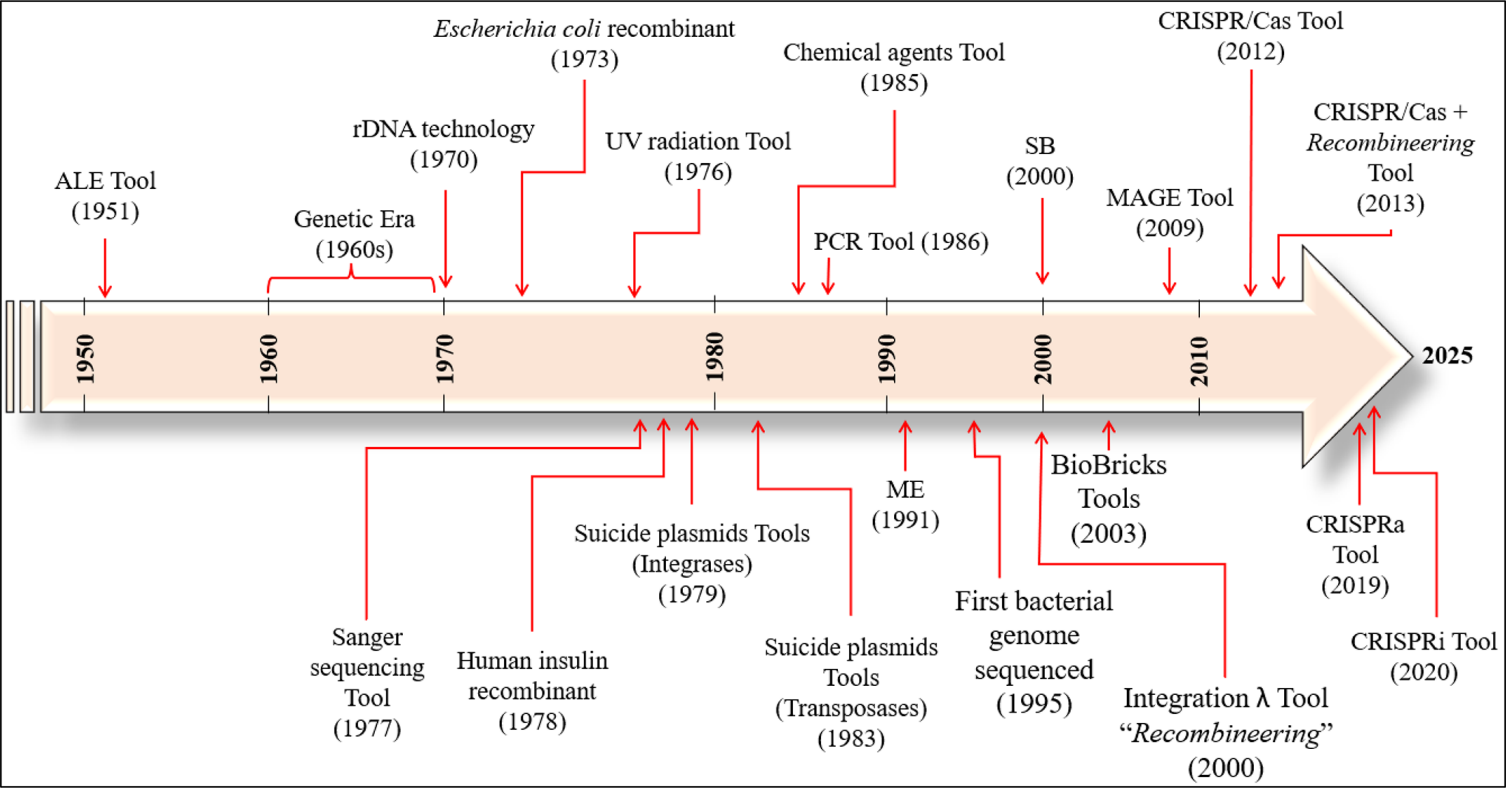
The timeline illustrates both the fundamental milestones in the development of genetic manipulation and the core genome editing tools in bacteria.

The history of genomic manipulation for the purpose of improving bacterial production is presented here chronologically (Fig. 1), highlighting the knowledge and efforts that have driven its evolution to the present day. In the 1960s, the ‘Genetic Era’ marked a turning point by enabling the development of procedures to generate ‘recombinant DNA’ (rDNA) [3], which later consolidated into ‘rDNA technology’ in 1970. In 1973, the first recombinant bacterium, *Escherichia coli*, was designed, capable of maintaining DNA and expressing exogenous proteins [4]. This advancement enabled the production of recombinant proteins such as human insulin, somatostatin, interleukin-2 and human growth hormone [5,8], revolutionizing industrial microbiology by allowing the synthesis of new products. Nevertheless, the biosynthesis of these and other compounds remained constrained by intracellular limitations, including complex regulations and by-product formation [9,10].

To address these limitations, researchers proposed manipulating entire cellular systems, giving rise to ME, defined by Bailey in 1991 as ‘the improvement of cellular capabilities through the manipulation of enzymatic, regulatory and transport systems using rDNA technology’ [11,12]. Built on diverse strategies, tools and knowledge, this field generated many products; however, significant challenges persisted, particularly the limited knowledge of gene sequences and functions, which hindered direct manipulation [13].

Faced with these limitations, research turned towards designing genomic editing strategies even without having the complete genome sequence, giving rise to the so-called random genomic engineering. Among the pioneering methodologies are adaptive laboratory evolution (ALE), implemented since 1951 [14]; ultraviolet (UV) radiation, applied in 1976 [15]; and the use of chemical agents, introduced in 1985 [16]. These tools, used as a complement to ME, enabled the optimization of the production of various bacterial metabolites and compounds. However, their application presented notable limitations, such as low transferability between species, the prolonged duration of selection processes and variability in the efficiency of results, which drove the search for more precise approaches.

In this context, the 1979 discovery of site-specific recombinases (or integrases) in temperate bacteriophages represented a major advance, as it facilitated the targeted integration of genetic fragments through the so-called suicide plasmids, although limited to predefined genomic sites [17,18]. Later, in 1983, transposon recombinases (or transposases) were incorporated, though these still integrated material into pre-established locations in the genome [19]. Together, these innovations marked the beginning of semi-random genomic engineering, a stage that not only expanded the possibilities for manipulating the bacterial genome but also enhanced the synthesis and productive improvement of new compounds. Over the years, the identification of recombinogenic proteins capable of integrating genetic material without relying on pre-established sites paved the way for the first rational tools [20,21]. This strategy allowed for specific edits in the bacterial genome, although it faced a critical limitation: the complex design and construction required for its implementation. While notable applications were reported, this restriction significantly limited its applicability across a broader range of bacterial species, both model and non-model.

DNA sequencing using the Sanger method (1977) [22] and polymerase chain reaction (PCR) in 1986 [23] laid the foundation for the first sequenced bacterial genome in 1995. This enabled the emergence of computational biology, which, combined with systems and network biology, led to SB by the late 1990s. SB introduced a systematic approach to designing and assembling molecular components, improving genomic tools [24,27]. In 2000, a tool capable of integrating and removing genetic material at any genomic site was developed, forming the basis of ‘Recombineering’ and marking the start of RGE, reducing editing times and enhancing bacterial metabolite production [28,30]. This also paved the way for next-generation tools like multiplex automated genome engineering (MAGE), 2009. However, it required further adaptations to reach its full potential in bacteria [31].

Among the most recent genome editing tools, the clustered regularly interspaced short palindromic repeats, associated with the Cas endonuclease (CRISPR/Cas) system, introduced in 2012, has stood out for its precision, versatility and robustness, surpassing previous methodologies [32,34]. Beyond complementing existing tools, CRISPR/Cas has been adapted to applications such as selective activation or repression of gene transcription [35,36]. Its use, independently or combined with other tools, has significantly advanced bacterial production. However, like any key genome editing tool, it has limitations, with off-target mutations being the main challenge. Continuous analysis, optimization and adaptation of CRISPR/Cas and other genome editing tools are essential to maximize their performance and adaptability.

In this way, unlike previous reviews focused exclusively on the technical foundations of genome editing or on specific applications of certain tools, this review critically integrates the historical evolution of these technologies with their impact on the design of production strains. Its distinctive contribution lies in connecting conceptual advances in genome engineering with practical examples of optimization. Thus, it not only synthesizes accumulated knowledge but also highlights the opportunities and challenges that shape the future direction of bacterial genome engineering.

## Metabolic engineering with random genome engineering tools to enhance bacterial productivity

**Fig. 2.**
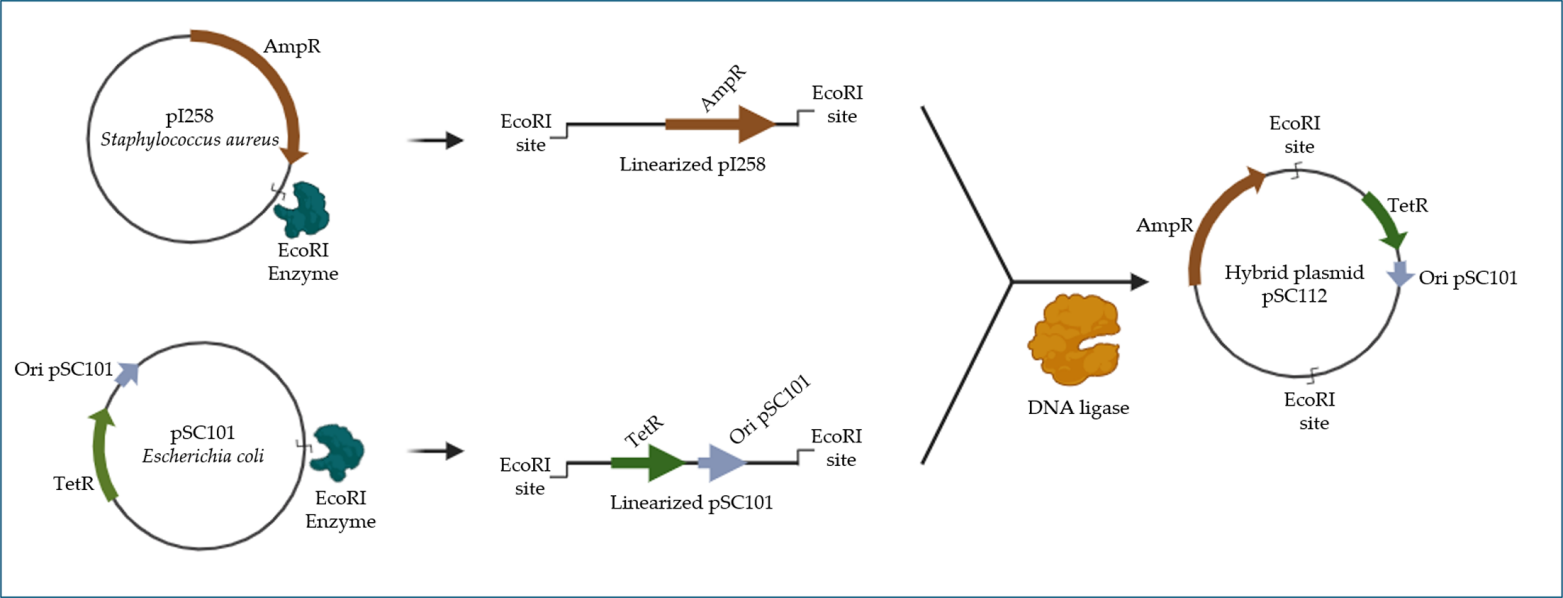
Shows that the pI258 plasmid (which resists ampicillin, AmpR) from *Staphylococcus aureus* and the pSC101 plasmid (which resists tetracycline, TetR) from *E. coli* were cut with the EcoRI endonuclease, creating straight pieces of DNA with sticky ends. After cleaning up each piece of DNA, they were joined together using *E. coli* DNA ligase, fixing the cut areas and creating a hybrid plasmid by linking both genetic materials into one unit, all done outside of a living organism. The resulting construction, pSC112, was transformed into competent *E. coli* cells. Thanks to the ‘ori pSC101’ origin of replication, the plasmid replicated and the transformation allowed the bacteria to express resistance to both antibiotics. The image was created using BioRender and is based on reference [4].

During the ‘Genetic Era’, the understanding of numerous molecular mechanisms in bacteria was greatly advanced, including the transformation [37], conjugation [38] and transduction [39] of genetic material. This knowledge made it possible to assemble unnatural DNA *in vitro*, later termed rDNA [3]. This procedure, colloquially known as ‘cut and splice’, emerged from the work of multiple researchers, including Luria and Human (1952) [40], Arber and Dussoix (1962) [41], Gefter [42], Oliveira and Lehman (1967) [43], Smith and Smith y Welcox (1970) [44], Danna and Nathans (1971) [45], Lobban (1972) [46], Boyer and Hedgpeth (1972) [47,48] and finally Mertz and Davis (1972) [49]. Thus, by mid-1973, Chang and Cohen developed the first recombinant organism using *E. coli*. Their work focused on constructing the first chimeric bacterial plasmid by fusing the *E. coli* plasmid pSC101 with the *Staphylococcus aureus* plasmid pI258 (Fig. 2) [4,50]. The successful transformation of this plasmid into the enterobacterium *E. coli* demonstrated its stability, as it retained exogenous DNA and enabled the expression of a *Staphylococcus aureus* protein that conferred resistance to the antibiotic used for selection.

This led the team headed by Bolívar, under the mentorship of Boyer, to develop in 1977 the first cloning vehicle for exogenous DNA: the plasmid pBR322. This plasmid proved highly versatile due to its small size, stability and the availability of multiple restriction sites for DNA insertion [51]. Subsequently, Goeddel and his team (1978) used it for the heterologous expression of the A and B chains of human insulin [5]. In 1982, the pharmaceutical company Eli Lilly optimized the production process, enabling the commercialization of insulin, thereby establishing it as the first recombinant product and the first approved by the US Food and Drug Administration (FDA) for human use [5]. This achievement spurred the synthesis of new recombinant proteins. Among the most notable examples are the production of somatostatin (1977) [6], human interleukin-2 (1984) [7] and human growth hormone (1986) [8], all obtained in *E. coli*. During the same period, the production of the enzyme *α*-amylase was also reported in *Bacillus subtilis*, whose optimization through rDNA technology enabled a 250-fold increase in production compared to the parental strain (1980) [52].

The rDNA technology revolutionized industrial microbiology by enabling the development of more efficient processes to produce recombinant proteins and both primary and secondary metabolites. Nevertheless, it was recognized that bacterial synthesis faced significant constraints that limited the production of these compounds, such as the extensive and complex regulations governing bacterial pathways, as well as the accumulation of by-products during synthesis, some of which proved toxic and compromised cell viability and, consequently, overall production [9,10]. To overcome these obstacles, an innovative strategy emerged: the manipulation of entire cellular systems, which gave rise to ME in 1991. This discipline became established as an approach focused on editing and optimizing native genetic systems, including catalytic enzymes, regulatory proteins and transporters, relying on rDNA technology [11,12]. Among the most widely employed techniques in ME are *in vitro* enzyme engineering, the inhibition of competing metabolic pathways and the overexpression of both homologous and heterologous genes [53]. For a more comprehensive review on ME, see [9,53].

ME established its foundations on a set of strategies, tools and fundamental knowledge that have enabled the identification, implementation and refinement of the most effective genetic manipulations to achieve desired modifications in bacteria. Among these are the appropriate selection of the bacterial host, the detailed characterization of metabolic pathways and their regulatory mechanisms, the use of molecular tools such as expression plasmids and inducible promoters, as well as the understanding of the stoichiometry and thermodynamics of cellular systems. These were further complemented by the implementation of more efficient transformation methods and the design of strategies to minimize the accumulation of by-products [11,13].

Model bacteria such as *E. coli* and *B. subtilis* have been pivotal for the development of ME; however, non-model bacterial chassis have also been explored and established, facilitating the synthesis of novel compounds and the optimization of previously known metabolites (Table 1) [54].

**Table 1.** Bacterial chassis explored in metabolic engineering

Organism	Tool	Result	Year	Reference
*E. coli* TC4	ME	Ethanol production	1987	[159]
*E. coli* JM101	ME	Haemoglobin expression	1988	[160]
*E. coli* FM4560	ME	Degradation of polychlorinated biphenyls	1989	[161]
*Klebsiella oxytoca* M5A1	ME	Ethanol production (40 g l^−1^)	1991	[162]
*Zymomonas mobilis* ZM4	ME	Ethanol production from starch (69.2 g l^−1^)	1991	[163]
*E. coli* AG1	ME	Production of 1,3-propanediol. (0.8 g l^−1^)	1991	[164]
*Corynebacterium glutamicum* KY10693	ME	Production of tyrosine (26 g l^−1^) or phenylalanine (28 g l^−1^)	1992	[165]
*E. coli* LS5218	ME	Production of poly-(3-hydroxybutyrate-co-3-hydroxyvalerate) (2.5 g l^−1^)	1992	[166]
*Clostridium acetobutylicum* ATCC 824	ME	Butanol/acetone production (23.2 g l^−1^)	1993	[167]
*E. coli*	ME	Antibody production	1994	[168]
*E. coli* BL21 (DE3)	ME	Production of chicken ovalbumin (0.0084 g l^−1^ of purified protein)	1995	[169]
*E. coli* DH5α	ME	Production of poly(3-hydroxybutyrate-co-4-hydroxybutyrate), 0.23 g l^−1^.	1997	[170]
*Corynebacterium glutamicum* BE	ME	Production of valerolactam (VL) (9.68 g l^−1^)	2023	[171]
*Bacillus licheniformis* DW2	ME	Production of ectoine (2 g l^−1^)	2025	[172]
*Pseudomonas chlororaphis* P3	ME	Phenazine-1,6-dicarboxylic acid production (6.45 g l^−1^)	2025	[173]

Although ME enabled the production and optimization of important products, significant limitations persisted, since many genes remained unknown in terms of their sequence and function and, therefore, could not be subjected to direct analysis or manipulation. In 1951, ALE was reported as an effective strategy to improve phenotypic traits, including production, by generating beneficial random mutations in the genome. This methodology relies on two main mechanisms: (1) the induction of modifications through gradual exposure (selective pressure) and (2) subsequent phenotypic screening to identify variants with adaptive advantages [14,55]. Exemplary cases are reported in [56,58]. Thus, although many of the modified genes remain unknown, the benefits of improving production with ALE promoted its use as a complement to ME.

In 1976, random mutation through UV radiation was introduced as another complementary strategy. This method randomly induced frameshifts, deletions, base substitutions and other genomic alterations [15], with documented applications in various cases [59,61]. Finally, in 1986, chemical mutagens became the last major tool to complement ME. Compounds such as N-methyl-N′-nitro-N-nitrosoguanidine and ethyl methanesulfonate (EMS) promoted genomic modifications by generating alkylated lesions that activated cellular repair mechanisms [16], a strategy that has also demonstrated significant applications [62,64].

**Fig. 3.**
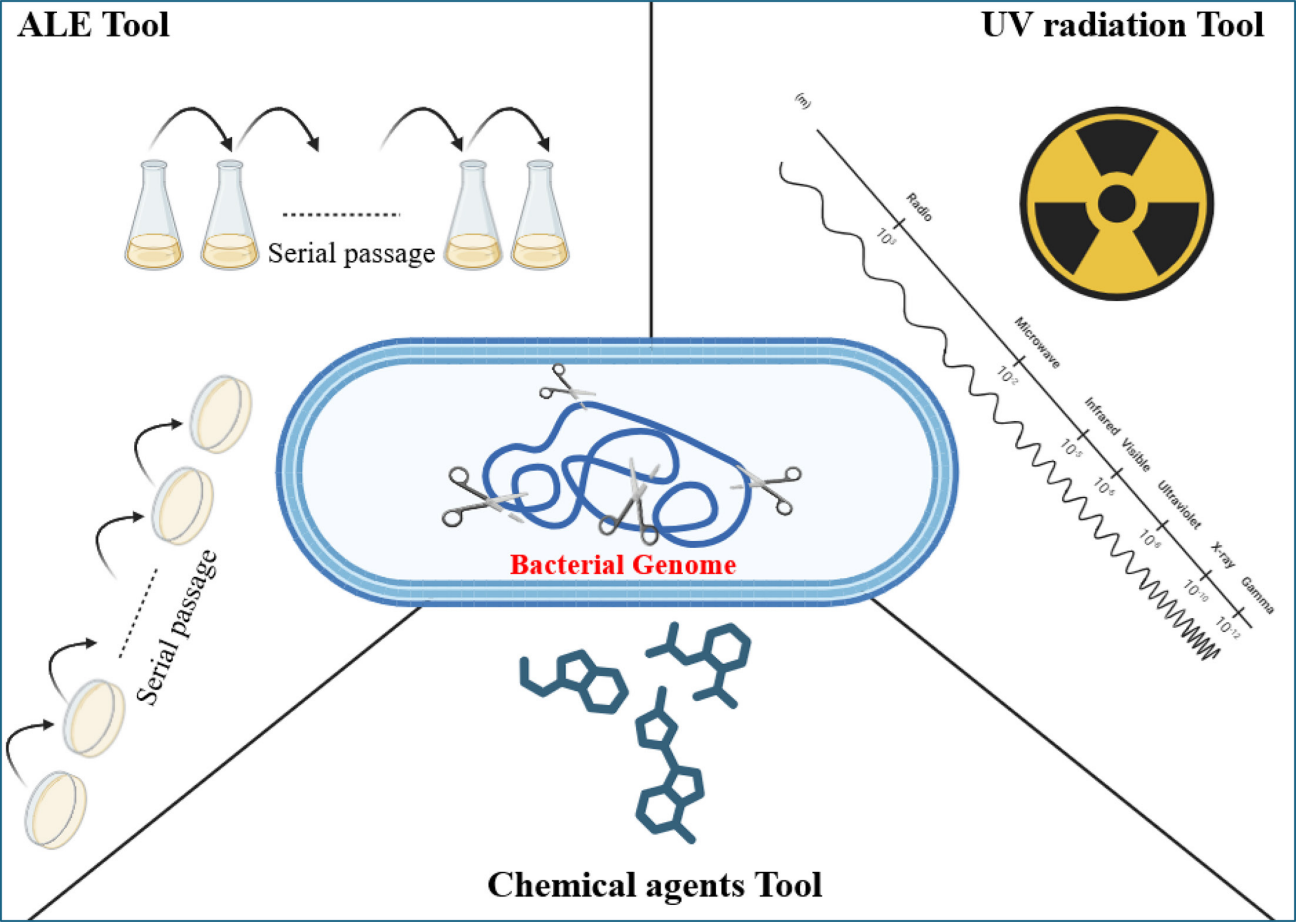
Random genomic editing tools.

Thus, alongside the rise of ME, genome engineering became established as a key component in bacterial improvement, enabling the optimization of compound production across different species (Table 2), although at this initial stage it relied primarily on random methodologies (Fig. 3).

**Table 2.** Representative examples of improved metabolite production using non-rational mutagenesis strategies in several bacteria

Organism	Tool	Result	Year	Reference
*Gluconobacter melanogenus* SPOI	UV	Enhanced production of 2-keto-l-gulonic acid (from 13 to ~60 g l^−1^)	1990	[174]
*Streptomyces* sp. P6621	UV	Enhanced cephamycin C production (from 1.27 to 2 g l^−1^)	1994	[175]
*Clostridium thermocellum* SS8	UV and nitrosoguanidine	Enhance production of ethanol (from 0.25 to 0.37 g g^−1^)	1996	[176]
*Bacillus* sp*.* MK716	EMS	Increased thermostable *α*-amylase production from 130 to 5300 U ml^−1^	1997	[177]
*Cellulomonas biazotea* NIAB 442	Gamma ray	Improved cellulase production: FPase 23.3 IU l^−1^, CMCase 52 IU l^−1^, *β*-glucosidase 15.2 IU l^−1^	1998	[178]
*Streptomyces fradiae* NRRL 2702	UV and nitrosoguanidine	Increased tylosin production	1999	[179]
*Amycolatopsis mediterranei* VA17/VA18	UV and nitrosoguanidine	Enhanced rifamycin B production (from 1.4 to 2.45 g l^−1^)	2000	[180]
*E. coli* JP01	ALE	Improved production of p-hydroxybenzoic acid (up to 6.2 g l^−1^)	2000	[181]
*Streptomyces avermitilis* 41445	UV, Ethidium bromide, EMS	High production avermectin B1b to 2.54 g l^−1^; parental strain produced 0.017 g l^−1^	2014	[182]
*Lactobacillus pentosus* CECT4023T	ALE	Enhanced lactic acid production (from 4.7 to 8.5 g l^−1^)	2019	[183]
*Bacillus coagulans* CC17B-1	ALE	Enhanced lactic acid production; 31.2 to 46.5 g l^−1^	2023	[184]
*B. subtilis* BS011	ALE	Improved tolerance of vitamin K (6 to 60 mg l^−1^ VK) to increase menaquinone−7 (MK-7) production from 0.053 to 0.0649 g l^−1^	2024	[185]
*Vibrio* sp*. VDHG*	ALE	Increased production of citramalate to 6.2 g l^−1^	2024	[186]
*Streptomyces geldanamycininus* FIM18-0592	UV	Improving geldanamycin production from 2.75 to 3.74 g l^−1^	2025	[187]

Although random methods have effectively complemented ME, significant limitations have emerged in many implementations, such as the difficulty of adapting to different bacterial species, the prolonged duration of selection processes, variability in efficiency and low specificity. However, these methods continue to be used today, as mutagenesis combined with selection remains a cost-effective procedure and, for the reliable short-term development of strains, often constitutes the most suitable strategy [65]. An emblematic example of its application is the pro220duction of *p*-hydroxybenzoic acid (*p*HBA) in *E. coli*. Liu *et al.* [66] developed a strain through ME that achieved a final concentration of 14.5 g l^−1^ of *p*HBA. Subsequently, after rounds of adaptive evolution applied to this strain, production increased to 21.3 g l^−1^, a 1.4-fold enhancement [66], thus demonstrating the potential of random genome editing to industrial strain improvement.

## Emergence of RGE tools: a foundation for the continuous development of strains with enhanced productivity

Although random genome engineering proved valuable for enhancing bacterial productivity, research efforts soon shifted toward the development of faster and more specific methodologies. In 1979, it was reported that the temperate λ phage encoded proteins capable of integrating its genetic material into the *E. coli* chromosome *in vivo*. This process was mediated by an integrase enzyme that promoted recombination between the attachment site in the phage DNA (*attP*) and the corresponding site in the bacterial genome (*attB*). Each integrase recognizes sequences; some act autonomously, while others require the participation of additional proteins encoded either by the phage itself or by the bacterial host [67]. The presence of homologous proteins has been reported in phages of bacteria such as *Haemophilus influenzae* [68], *Mycobacterium smegmatis* [69], *Salmonella enterica* [70] and *Pseudomonas putida* [71].

By 1983, another semi-random genome editing strategy had been reported: the use of transposons. These were described as mobile DNA elements capable of integrating *in vivo* into specific regions of both bacterial genomes and plasmids [19]. Unlike viral integrases, transposons employ recombinases known as transposases, which are encoded by the elements themselves. These enzymes recognize short sequences of 4 to 10 bp located at the ends of the transposon, termed terminal inverted repeats. The complex formed by transposases and the transposon ends, known as the transposome, recognizes these sites and facilitates both the integration and excision of the element [72].

Both integrases and transposases played a key role in the development of tools for compound production. Following the discovery of novel integrase variants in *Staphylococcus aureus* and *Actinobacteria*, the use of suicide plasmids carrying a selectable marker alongside *attP* sites was consolidated, complemented by a second plasmid responsible for expressing heterologous integrases. Integration occurred at the endogenous *attB* sites of the bacterium and could be readily selected [73]. Thanks to this strategy, in 1991, a live attenuated vaccine was produced from the *B. subtilis Calmette–Guérin* strain, capable of expressing antigens against *Mycobacterium tuberculosis* [74]. In *Pseudomonas aeruginosa*, a more efficient version was designed, based on the ΦCTX recombinases and their integration sites, which also included a tetracycline resistance marker and a multiple cloning site. This construction displayed considerably higher recombination frequencies [75]. In parallel, it has been reported that the integration of transposons into promoter regions can enhance gene expression, as observed with the overexpression of the *ampC* gene, which encodes *β*-lactamase, in *Acinetobacter baumannii*, thereby increasing resistance to cephalosporins. Similarly, in *Acinetobacter bereziniae*, the insertion of a transposon into a promoter region improved resistance to carbapenems.

Additionally, the portability of these elements was demonstrated by cloning them into plasmids. A representative example is the pBAMD vector, which carries a mini-Tn5 capable of integrating genes into the genome of Gram-negative bacteria. Among its most notable applications is the integration of the polyhydroxybutyrate biosynthetic pathway from *Cupriavidus necator* into *E. coli*, enabling the overproduction of this compound. For a more detailed analysis of the use of transposons in bacterial improvement, see [72].

One of the key studies that drove the search for more specific strategies for chromosome editing was based on the ability of *Saccharomyces cerevisiae* to integrate linear plasmids into any region of its genome (1983) [76]. Its intrinsic capacity to perform homologous recombination, using DNA with homologous regions at the ends as a substrate for integration, served as a model to identify equivalent mechanisms in bacteria. Initially, this research focused on *E. coli*, where it was observed that linear DNA could not be transformed due to the action of exonucleases that degraded the genetic material, preventing homologous recombination. To overcome this limitation, it was necessary to generate host strains with a recombinogenic genetic background capable of facilitating the integration of linearized plasmids [77]. In 1989, Russell *et al.* developed a *recD* mutant strain through phage-mediated transduction, which they demonstrated had a modified genetic background that could be more stable and efficient for DNA integration via linearized plasmids [20]. That same year, Hamilton *et al.* [21] proposed a method for site-specific gene integration without the need for linearized plasmids, although its implementation required complex steps to construct plasmids tailored to the target mutation site [21]. In 1996, Metcalf and collaborators improved this approach by incorporating suicide markers with the R6Kγ origin of replication, increasing precision and reducing false positives among the resulting mutants [78]. It was not until the late 1990s that Pósfai developed a simpler and more efficient technique for gene replacement. Their method employed a non-specific suicide plasmid (i.e. different from those used by integrases or transposases) carrying the selectable construct along with a recognition site for the *Saccharomyces cerevisiae* meganuclease I-SceI. As a result, it became possible to obtain bacteria with and without the desired mutation, requiring only PCR-based detection steps [79].

In other bacteria, linear non-specific suicide plasmids with specific homologous arms have been developed to generate targeted mutations. In *Yersinia enterocolitica*, a suicide plasmid was designed with sequences homologous to the *yadA* gene, whose chromosomal deletion reduced the bacterium’s resistance to antibodies [80]. A similar approach in *Citrobacter freundii* demonstrated that deleting the *eaE* gene prevents colonization of the colon in mice [81]. Likewise, in *Rhizobium meliloti* and *Rhizobium leguminosarum*, a non-specific suicide plasmid was constructed with homologous arms targeting the *recA* gene in both species. Its chromosomal integration and subsequent *recA* replacement resulted in recombination-deficient phenotypes, while the insertion remained stable over multiple generations [82]. In the same way, researchers removed the *recA* gene in *Corynebacterium glutamicum* and *Brevibacterium lactofermentum* using a special plasmid. It caused less ability to recombine DNA and made them more sensitive to UV light, mitomycin C and methyl methanesulphonate (MMS) [83]. Finally, in *Shigella flexneri*, the construction of a non-specific suicide plasmid demonstrated that the *mxiD* gene is essential for bacterial infection and its ability to induce keratoconjunctivitis in guinea pigs [84]. Despite these advances, significant limitations persisted, such as the requirement for long homologous arms, the prolonged duration of procedures and low rates of successful recombination. These constraints demonstrated that, although functional, the method was not sufficiently practical for routine applications.

With the establishment of Sanger DNA sequencing in 1977 [22] and the introduction of PCR by Mullis in 1986 [23], it became possible to obtain the first complete genomic sequence of a bacterium: *H. influenzae* [85]. The availability of genomic sequences promoted the study and compilation of information on numerous genes involved in regulation, metabolism, transport and other cellular processes that had previously remained unknown [86]. In the case of *E. coli* K-12 [87], much of this information began to be stored in electronic databases, such as RegulonDB [88], EcoCyc [89] and Kyoto Encyclopedia of Genes and Genomes (KEGG) [90]. Over time, the number of available bacterial genomes continued to grow rapidly; species such as *B. subtilis* [91], *Helicobacter pylori* [92], *Aquifex aeolicus* [93], *M. tuberculosis* [94] and *Thermotoga maritima* [95] were added to this expanding repertoire, establishing a fundamental database that would drive the development of RGE tools in ME.

By the late 1990s, computational biology emerged as a discipline aimed at managing this information and strengthening electronic databases. It enabled the study and reconstruction of complete genomes, the design of genetic and metabolic networks and the prediction of protein structures, among other applications [96,98]. Its integration with systems biology [99] fuelled the rise of SB in the early 2000s, facilitating the design and construction of new genetic features in bacteria and promoting the development of more precise and efficient genomic tools, thereby expanding the possibilities for productive optimization in bacteria [100].

During the early stages of SB, its rational and precise approach enabled the introduction of so-called ‘BioParts’ or ‘BioBricks’ (2003): modular units composed of interchangeable molecular parts, either individually or assembled into sets, which facilitated the construction of functional modules and complex systems with specific applications [101,102]. Thanks to these advances, BS evolved toward a more systematic, predictable and standardized approach, allowing the design and construction of increasingly sophisticated genetic circuits that were functional and adaptable to diverse applications. In the context of ME, these developments facilitated the assembly of complete metabolic pathways and improved regulation and expression during compound production.

**Fig. 4.**
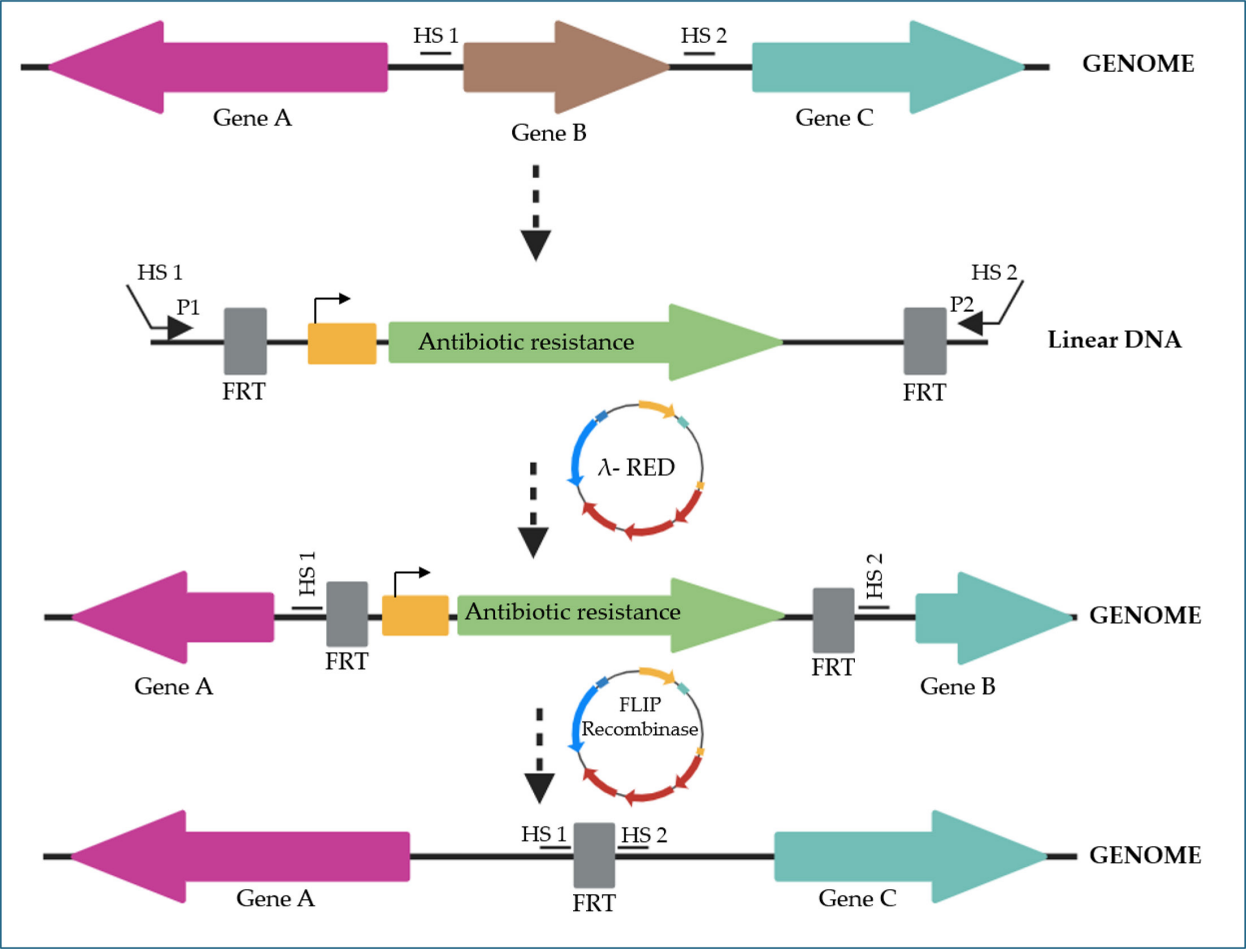
The λ integration tool first used the genetic sequence of the location where new genetic material would be added (gene B in this case) to create the oligonucleotides that include homology sites 1 (HS1) and 2 (HS2), as well as the sites that connect with the ‘antibiotic resistance gene’, P1/P2. After obtaining and purifying the linear DNA through PCR, the double-stranded DNA (dsDNA) was introduced into bacteria containing the λ-RED plasmid, which had been previously cloned with the Exo, Bet and Gam proteins. Following a recovery phase, the cells successfully integrated the resistance gene precisely between the HS1 and HS2 regions, thereby ‘replacing’ gene B in the *E. coli* chromosome. To remove the resistance gene, a second plasmid was utilized, which was introduced into the mutated strain. This plasmid had been cloned with an FLP recombinase, which recognized the FRT sites (inserted during the construction of the marker gene), excising that fragment from the chromosome and leaving behind a small scar (FRT site). The image was adapted from reference [28] and modified using BioRender.

By the mid-2000s, the robust and precise approach of SB enabled Datsenko and Wanner to develop a tool capable of integrating, *in vivo*, a marker gene into any region of the *E. coli* chromosome. Their strategy relied on linear DNA with short homology arms (30–50 bp), processed by the λ phage proteins Exo, Beta and Gama, expressed from an auxiliary plasmid, thereby eliminating the need for a recombinogenic background and making the system applicable across different strains. The same plasmid also carried the gene encoding the FLP recombinase from *Saccharomyces cerevisiae*, whose role was to excise the antibiotic resistance marker after recombination. Although this process left a scar in the genome, the ability to remove the marker was essential (Fig. 4) [28]. This system proved more efficient than the Rac phage RecE/RecT method (1998) [103] and enabled the stable generation of specific mutations throughout the genome. Among the most notable achievements were the deletion of genes to enhance the production of pyruvate [104], lycopene [105], lactic acid [106] and succinic acid [107], as well as the construction of the KEIO collection, in which each individual *E. coli* gene was systematically deleted [29].

Its application as a tool for bacterial genome rational editing consolidated the approach now known as the ‘Recombineering’ technique [108,109]. Nevertheless, like any technology, it exhibited limitations that restricted its implementation across a broader range of bacteria, which in turn drove the development of various solutions. For example, specific modifications to *Recombineering* systems have been implemented to adapt them to other Gram-negative bacteria closely related to *E. coli*, such as *Serratia*, *Vibrio* and *Klebsiella*. These improvements have increased the stability of transformed DNA, optimized the expression of recombinase proteins and refined experimental conditions to facilitate homologous recombination. In *Salmonella enterica*, the *Saccharomyces cerevisiae* endonuclease I-SceI was added to create breaks in the DNA, making it easier to remove sections without leaving marks, change specific DNA sequences and join parts of the genome at the beginning and end [110]. In *P. aeruginosa*, several additional adaptations were implemented, including (I) extending the homologous regions in PCR fragments from 50 to 100 bp to enhance mutation efficiency, (II) developing a plasmid that enabled controlled and non-toxic expression of λ proteins and (III) eliminating genomic scars [111]. Even in *E. coli*, improved versions of the Datsenko and Wanner tool were developed, such as those proposed by Kuhlman and Cox [112], as well as by Tas *et al.* [113].

Additionally, innovative recombination systems adapted to different bacterial hosts were developed, enabling their implementation in *Lactobacillus*, *Mycobacterium*, *Pseudomonas*, *Burkholderia* and others [108,109]. Notable among these is the allele-coupled exchange system, developed for *Clostridium acetobutylicum*, an anaerobic bacterium with high potential in biofuel production. This technique enabled the rapid, precise and efficient integration of long DNA fragments directly into the chromosome, facilitating the modification of multiple loci without the need for counterproductive selection steps, thus optimizing industrial strain engineering [114]. Another important system is flexible recombineering using integration of *thyA* (FRUIT), applied in *S. enterica*. This system allows precise genetic modifications without leaving ‘genetic scars’, meaning no selection markers remain after the editing process. By using the *thyA* gene as a marker that can be removed later, FRUIT makes it possible to add small changes, remove genes and insert useful sequences like epitopes or promoters, allowing for precise adjustments in specific areas of enteric bacteria [115]. Finally, single-stranded annealing protein-independent recombination has been employed in Gram-positive bacteria such as *Brevibacterium lactofermentum* and *C. glutamicum*. This strategy allows the efficient incorporation of ssDNA oligonucleotides without the need to express auxiliary recombinase proteins, simplifying its application. Since it does not require heterologous genes or complex systems, this tool is particularly useful for the engineering of generally recognized as safe (GRAS) bacteria, which are widely used to produce amino acids, vitamins and other relevant compounds [116].

Each of these RGE tools has significantly contributed to improving performance in the production of a wide variety of metabolites in bacterial systems. Thanks to their implementation, it has been possible to optimize metabolic pathways, increase gene expression efficiency and overcome limitations associated with the traditional design of strains. These advancements are reflected in the notable increases in the synthesis of high-value compounds, as summarized in Table 3.

**Table 3.** Applications for homologous recombination in bacterial engineering

Organism	Tool	Result	Year	Reference
*Synechocystis* sp. PCC 6803	Homologous recombination (double crossover)	Increase carotenoid production (from 0.0016 to 0.0025 g l^−1^)	2000	[188]
*E. coli* K12	Homologous recombination (λ Red)	Increased lycopene production (from 11.5 to 23 g l^−1^)	2005	[189]
*E. coli* MC1061	Homologous recombination (λ Red)	Increased *β*-carotene production to 0.006 g g^−1^ dry cell weight	2006	[190]
*B. subtilis* RH33	Homologous recombination (single crossover)	Increase in riboflavin production (from 0.03 to 0.045 g g^−1^ dry cell weight)	2007	[191]
*E. coli* ATCC 8739	Homologous recombination (λ Red and double crossover)	Increased succinate production	2008	[192]
*Bacillus thuringiensis* sp. kurstaki Cry⁻B	Homologous recombination (λ Red)	Overexpression of Recombinant *cry1Ac* and *CDEP2* proteases	2009	[193]
*B. subtilis* PY	Homologous recombination (single crossover)	Increased riboflavin production (from 0.54 to 1 g l^−1^)	2010	[194]
*Brevibacterium flavum* *JV16*	Homologous recombination (single crossover	Increase l-valine production (from 8.5 to 34.4 g l^−1^)	2012	[195]
*E. coli* K4	Homologous recombination (FRT/FLP recombination cassette integrative)	Increased production of capsular polysaccharide (from 0.124 to 0.283 g l^−1^)	2013	[196]
*E. coli* TBW20108	Homologous recombination (λ Red)	Coenzyme Q10 is produced at 0.0774 g g^−1^	2014	[197]
*E. coli* ATCC 8739	Homologous recombination (λ Red)	Enhanced phenol production (from 0.0017 to 9.5 g l^−1^).	2015	[198]
*B. subtilis* *THY-7*	Single-crossover homologous recombination (RecA proteins)	Increase surfactin production (from 0.55 to 9.7 g l^−1^).	2017	[199]
*C. glutamicum* ATCC14067	Homologous recombination (RecET-Cre/loxP system)	Increase l-leucine production (from 0.07 to 14 g l^−1^)	2020	[200]
*Streptomyces avermitilis* A229	Homologous recombination (double crossover)	Increase avermectin B1a (from 6.447 to 9.613 g l^−1^)	2022	[201]

However, as noted by Elmore *et al.*, these tools are generally limited to the integration of one or two DNA fragments, function in a restricted number of hosts, rely on plasmids that replicate in the host and often leave selectable markers or scars in the chromosome [117]. Yan *et al.* agree with Elmore that these tools exhibit a limited host range. They point out that, unlike model bacteria, non-model strains lack sufficient information and robust genomic tools, which restrict their development and application. Advancing in this field requires substantial efforts, including systematic studies on the utilization of different substrates and the production of multiple compounds, essential steps for enabling these non-model bacteria to evolve into model organisms [118].

For example, *Saccharopolyspora spinosa* is a non-model, Gram-positive bacterium belonging to the phylum *Actinobacteria*, currently used for the biosynthesis of the insecticide spinosad. However, genomic editing of this species has posed significant challenges. Early strategies relied on UV mutagenesis, and the resulting strains were subjected to adaptive evolution processes to enhance productive traits. Using this approach, a strain was obtained with a production of 0.547 g l^−1^, representing a 436% increase over the parental strain [119]. More recently, a 2025 study successfully reported genomic editing using CRISPR/Cas, increasing spinosad production from 0.309 to 0.693 g l^−1^ [120]. Despite these advances, *S. spinosa* continues to present major challenges, including a low transformation efficiency, a GC-rich genome and the limited availability of tools for its manipulation.

In recent years, research has focused on exploring new bacterial chassis, with the following examples standing out: *Mycoplasma genitalium*, with an extremely small genome of 580 kb [121]; *Rhodococcus* sp., a producer of secondary metabolites and bioactive steroids [122]; *Streptomyces clavuligerus*, a generator of secondary metabolites such as clavulanic acid [123]; *Alteromonas* sp., capable of biodegrading polycyclic aromatic hydrocarbons [124]; *Shewanella oneidensis*, capable of generating bioelectricity [125]; *Vibrio natriegens*, with a rapid growth rate, ideal for implementing genetic tools [126]; *E. coli* TOP10, which produces higher concentrations of phytohormones [127]; and *Burkholderia sacchari*, used for biopolymer production [128]. For a more comprehensive review on the use and exploitation of novel bacterial chassis, see [54].

## New RGE tools: synthetic evolution and CRISPR/Cas towards faster and more accurate strategies for enhancing bacterial

In the last two decades, advances in the understanding of DNA and RNA have profoundly transformed the tools of genome engineering. This progress has strengthened both ME and genome engineering in model organisms and in organisms that have sought to become models and has enabled the development of methodologies to generate multiple deletions, insertions and genetic replacements. Initially, adaptive evolution proved essential to obtain strains more robust and better adapted to productive conditions; nevertheless, its random nature carried the risk of altering genes crucial for bacterial viability. The next generation of approaches to introduce multiple mutations arose with RGE, based principally on the *Recombineering* technique [129].

MAGE was introduced by Wang *et al.* in 2009 as the first tool capable of performing multiple genome editing. Initially reported in *E. coli*, this technique relies on the use of single-stranded DNA (ssDNA) oligonucleotides, specifically designed and synthesized for target sites, which are incorporated into the genome through homologous recombination proteins. In this way, small modifications such as substitutions, deletions or insertions can be generated in different chromosomal regions in a highly efficient and automated manner. In their work, the authors also demonstrated the potential of MAGE by applying it to optimize the 1-deoxy-d-xylulose-5-phosphate pathway, achieving the simultaneous modification of 20 endogenous genes and an increase in lycopene production. This breakthrough gave rise to the concept of ‘synthetic evolution or accelerated evolution’ a multiple-rational chromosome-scale editing strategy that differs from the random nature of adaptive evolution [130,131].

After this tool presented several limitations, including the requirement to pre-modify the host bacterium, which in turn led to the accumulation of off-target mutations. In practice, this involved altering the DNA repair machinery during ssDNA recombination to prevent the restoration of original sequences and enable editing. However, the deletion of mismatch repair systems significantly increased nonspecific mutations, making this strategy less suitable. Consequently, alternative solutions have been proposed, such as the redesign of oligonucleotides, the manipulation of repair machinery or its transient suppression, which are considered more promising approaches. Even with these improvements, the most significant restriction has been the system’s applicability, which remains largely confined to a small number of bacteria closely related to *E. coli*. The challenges include both the identification of recombinogenic proteins homologous to Redβ (of *E. coli*) and the development of efficient ssDNA transformation methods in other microorganisms. In this regard, bioinformatic analyses have enabled the identification of homologous enzymes that allow multiplex mutagenesis, as demonstrated in *P. aeruginosa*. Moreover, computational tools serve as a key aid in oligonucleotide design, considering parameters such as homology length and, in particular, the guanine-cytosine (G-C) content of the target genome. This consideration is crucial to avoid the formation of secondary structures in oligonucleotides that could hinder their chromosomal integration. For a more in-depth understanding of the fundamentals and applications of MAGE, see [132].

In 2011, conjugative assembly genome engineering was introduced to enable large-scale genome assembly through bacterial conjugation, allowing the transfer and fusion of engineered genomic segments between strains and overcoming the size and modification limits of MAGE. In 2012, co-selection MAGE improved efficiency by incorporating positive co-selection with selectable markers, enriching cell populations carrying multiple simultaneous modifications and facilitating genome-scale metabolic engineering. In 2016, transient mutator MAGE combined transient mutator expression with MAGE, increasing mutation rates only during recombination to enhance genetic diversity without compromising long-term stability, a key requirement for industrial use. That same year, a portable plasmid-based platform (pORTMAGE), expanded the applicability of MAGE to non-model strains, broadening its impact across diverse biotechnological contexts [133]. MAGE has also been implemented in *C. glutamicum* and *B. subtilis*, although significant adaptations were required due to differences in recombination mechanisms, transformation efficiency and the response to genome editing systems compared to *E. coli* [114]. Notably, in 2015, the tool simultaneous multiplex genome engineering (SMGE) was developed in *B. subtilis* [134], and it was subsequently used to optimize the l-tyrosine biosynthetic pathway to enhance the production of resveratrol (2021), a polyphenol of pharmacological and nutritional interest (2021) [135].

**Fig. 5.**
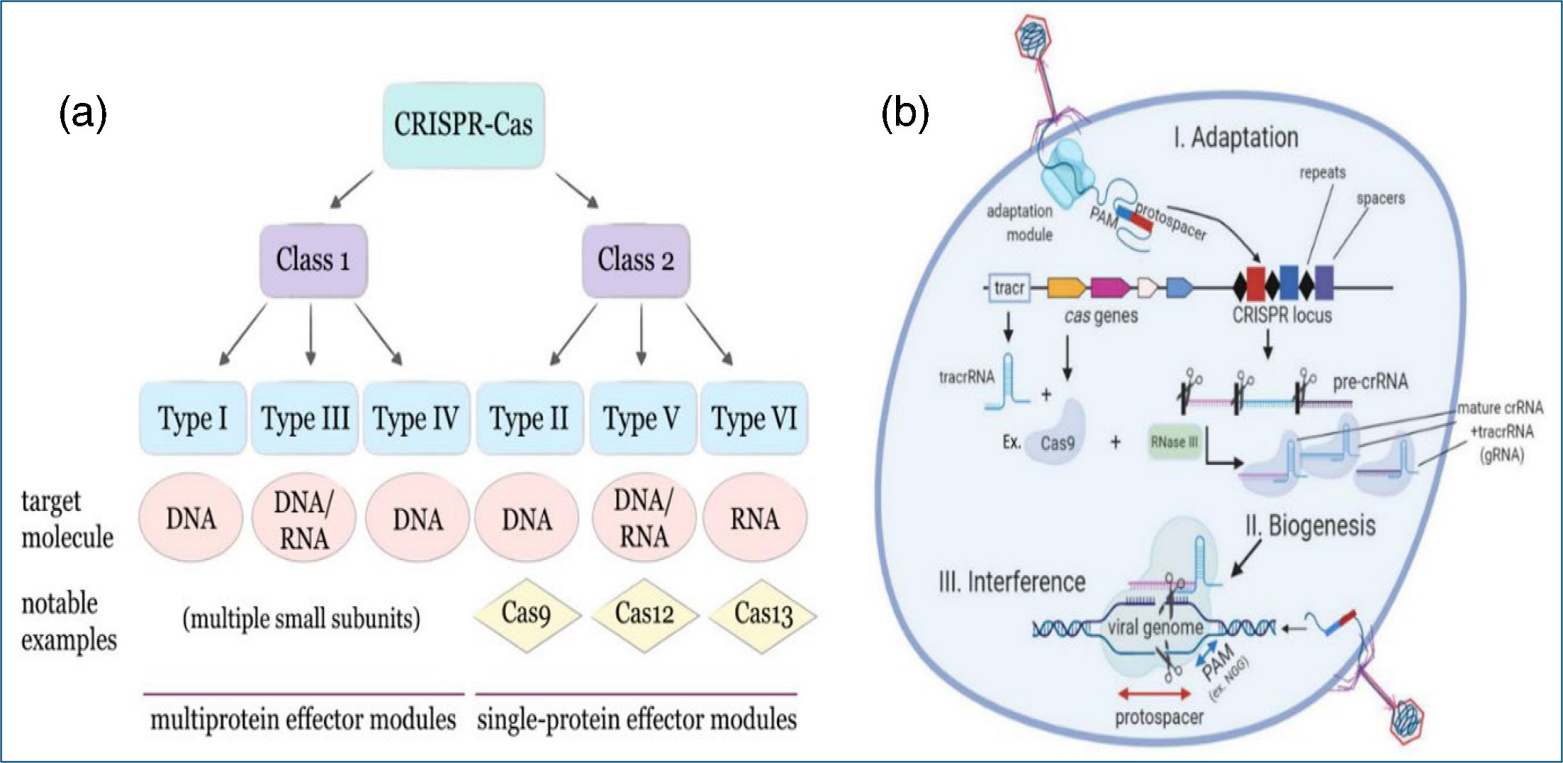
(**a**) CRISPR-Cas systems are divided into two classes (class 1 and class 2) and six main types (types I to VI), differentiated by their protein architecture and mechanisms of action. (**b**) The response consists of three stages: (I) adaptation – the insertion of foreign genetic material into the bacterial cell, followed by its recognition and cleavage by proteins from the adaptation module (black box, PAM-protospacer adjacent motif) to generate spacers. (II) Biogenesis: Transcription of the CRISPR locus. The resulting pre-CRISPR RNA (pre-crRNA) is processed by the Cas9/RNase III complex, which recognizes repeated sequences. The process produces mature crRNAs that subsequently pair with trans-activating CRISPR RNA (tracrRNA) to form guide RNA (gRNA). (III) Interference: When the genetic material is put back into the cell, if it has the right protospacer and PAM sites, the Cas/gRNA complexes recognize it and cut it. Images were adapted from [138,139].

In 2012, a tool that would revolutionize genomic editing in scientifically and industrially relevant bacteria emerged: CRISPR/Cas. Like the previously described strategies, this tool was developed based on accumulated knowledge from earlier research. Chronologically, in 1987, Ishino and collaborators [133] reported the presence of unusual repetitive sequences in the *E. coli* genome. Later, in 2002, Mojica and collaborators demonstrated that these sequences were not unique but derived from pre-existing fragments, leading to the hypothesis that they might be involved in adaptive immunity against foreign DNA [136]. In 2003, Jansen and collaborators identified, for the first time, genes associated with CRISPR, called ‘Cas’ genes, located near CRISPR loci in various bacteria. Sequence analysis revealed conserved motifs typical of helicases and nucleases, suggesting that the encoded proteins play a direct role in DNA manipulation. They also classified these proteins into two main modules: (1) the adaptation module, responsible for incorporating new fragments of foreign DNA as spacers, and (2) the effector module, which recognizes and cleaves foreign DNA during the interference phase [137]. Today, a broader classification encompassing all known CRISPR-Cas systems is recognized, as illustrated in Fig. 5a.

Until 2008, Brouns identified RNA molecules that guide the effector proteins of the CRISPR system, called CRISPR RNA (crRNA). During transcription of the CRISPR locus, precursors known as pre-crRNA are generated, which are subsequently processed into individual functional fragments, each containing a specific spacer that directs the immune response (Fig. 5b) [138,139]. By 2012, Gasiunas and his team demonstrated that only 20 bp of RNA were required for the CRISPR/Cas system to be functional [140]. That same year, Sapranauskas and collaborators conducted one of the first experimental studies validating the system’s ability to cleave DNA by introducing the endogenous *Streptococcus thermophilus* system into *E. coli* along with its natural crRNA, conferring resistance to certain viruses and plasmids [141].

Ultimately, Jinek *et al.* made a decisive advance by showing that crRNA could combine with another RNA molecule, trans-activating crRNA, to form a functional complex in bacteria. They also demonstrated that these two molecules could be fused into a single hybrid RNA, called single-guide RNA (sgRNA), capable of directing the Cas9 protein specifically to the target DNA, resulting in its cleavage [142]. This discovery laid the foundation for the development of CRISPR/Cas as a flexible and precise genome-editing tool in bacteria.

The number of studies on genetic modifications in bacteria has increased over time. One of the most comprehensive works was published by Cho and Shin [143], which documents the most notable achievements of modified bacteria from 2014 to 2018. Some examples include *Bacillus licheniformis* (gene deletion and insertion in the chromosome), *Streptomyces lividans* (gene insertion) and *P. putida* (genome editing), among others. Another study, focused primarily on lactic acid bacteria, was published by Roberts and Barrangou in 2020. Some of their examples are *Lactobacillus casei* (which has a quick method for changing its genome), *Lactococcus lactis* (which uses a system to help with DNA changes and a way to select against it using CRISPR/Cas9) and *Streptococcus mutans* (which uses CRISPR/Cas9 to remove harmful genes) [144]. In turn, Vento and collaborators (2020) compiled in an extensive table various bacteria that have been edited using this system, highlighting among them *Bacillus smithii*, *Clostridium beijerinckii*, *Enterobacter aerogenes*, *Tatumella citrea* and *Yersinia pestis* [145].

Editing efficiencies ranging from 50 to 95% have been observed, surpassing those achieved with *Recombineering*, which typically do not exceed 40%. This performance largely depends on the bacterial chassis, the transformation method employed and the CRISPR/Cas variant used [139].

Thus, this system has become a key tool for the generation of strains of great research interest; however, its use has been more prominent in the development of industrial strains, complementing ME to optimize the production of metabolites of interest, as detailed in Table 4.

**Table 4.** Recent applications of CRISPR/Cas-based technologies for bacterial productivity improvement

Organism	Tool	Result	Year	Reference
*E. coli* BL21 (DE3)	CRISPRi/dCas9	Increased flavonoid (2S)-naringenin production to 0.42 g l^−1^	2015	[202]
*E. coli* EcKan	CRISPR/Cas9 and λ-Red *Recombineering*	Increased the β-carotene production to 2.0 g l^−1^	2015	[203]
*C. glutamicum* ATCC 13032	CRISPRi/dCas9	Increased amino acid production	2016	[204]
*E. coli* ATCC 9637	CRISPR/Cas9 and CRISPRi	Increased 1,4-butanediol production to 1.8 g l^−1^	2017	[205]
*Synechocystis* sp. PCC 6803	CRISPRi/dCas9	Increased fatty alcohol production compared to the WT strain	2018	[206]
*Klebsiella pneumoniae*	CRISPRi/dCas9	Increased production of 3-hydroxypropionic acid to 36.7 g l^−1^	2018	[207]
*Halomonas* sp.	CRISPR/Cas9	Increased production of hydroxybutyrate (3HB) and 3-hydroxyvalerate (3HV)	2018	[208]
*B. subtilis*	CRISPRi/dCas9	Increased production of N-acetyl glucosamine	2018	[209]
*B. subtilis* REG19	CRISPR/Cas9	Increased production of β-galactosidase to 8.36 U mg^−1^	2019	[210]
*Synechococcus elongatus* UTEX 2973	CRISPR/Cas9	Increased free fatty acid production from 0.002 to 0.0122 g l^−1^	2021	[211]
*Streptomyces coelicolor* M1152	CRISPR/Cas9	Increased production of oviedomycin to 0.67 g l^−1^	2023	[212]
*E. coli* MG1655	CRISPR/Cas9	l-Threonine improvement production to 127.3 g l^−1^	2023	[213]
*Bacillus amyloliquefaciens* HZC9n	CRISPR/Cas9n	Improvement in heme production to 0.011 g l^−1^	2024	[214]
*Streptomyces avermitilis*	CRISPRi/dCas9	Increased production of avermectin to 0.452 g l^−1^	2024	[215]
*B. subtilis* BS1	CRISPR/Cas9	Increase production of *α*-amylase 57.9-fold	2024	[216]
*C. glutamicum*	CRISPRa/i bifunctional via dCpf1–SoxS	Increase production of shikimic acid and l-serine	2024	[217]
*Saccharopolyspora spinosa*	CRISPRi/dCas9	Increased production of spinosad to 0.633 g l^−1^	2025	[218]
*E. coli* W3110	CRISPR/Cas9	Increased production of l-tryptophan to 34.1 g l^−1^	2025	[219]
*E. coli* MG1655	CRISPR/Cas9 and λ-Red *Recombineering*	Increased production of isobutanol to 48.37 g l^−1^	2025	[220]

Several adaptations of the CRISPR/Cas tool have been developed, enabling not only precise genome editing but also DNA insertion through its combination with the *Recombineering* technique (2013) [146,147]. In addition, variants aimed at transcriptional regulation have been implemented, such as CRISPR activation (CRISPRa) in 2019 [148] and CRISPR interference (CRISPRi) in 2020 [149]. Table 4 shows examples of how these tools can be used to improve bacterial production.

For the first case, Jiang *et al.* integrated the CRISPR/Cas9 system with the *Recombineering* technique in *E. coli*, enabling the induction of double-strand breaks (DSBs) at virtually any genomic region. As DSBs are lethal, only cells that successfully incorporate DNA (plasmid, PCR-derived fragment or synthetic oligonucleotide) as a repair template can survive. This approach achieved success rates up to 65%, making it the most efficient bacterial genome engineering method to date, and has since been adapted for other genera, including *Streptococcus*, *Lactobacillus*, *Streptomyces* and *Clostridium* [146,147]. In CRISPRa, the dCas9–sgRNA–activator complex interacts with the target promoter, recruiting RNA polymerase and promoting transcription. However, bacterial CRISPRa systems show relatively low efficiency and are highly dependent on the precise spacing between the binding site and promoter [148]. Peters *et al.* highlight key advantages of CRISPRi: (I) rapid target reprogramming by altering the 20-nt sgRNA region (though large-scale library design requires computational tools); (II) scalability, enabling pooled cloning of thousands of sgRNAs; (III) inducibility, allowing partial repression of essential genes and improving stability in libraries targeting non-essential ones; and (IV) multiplexing, enabling simultaneous repression of multiple genes with several sgRNAs. Its main limitation is interference with downstream gene expression in polycistronic operons, as catalytically dead Cas9 (dCas9) blocks RNA polymerase elongation [35,149]. The review by Call and Andrews includes a comprehensive table summarizing bacterial strains modified using CRISPRi/a systems [150].

The effective implementation of the CRISPR/Cas system in recipient bacteria still faces several technical challenges that require specific optimization. For instance, the toxicity associated with Cas protein overexpression can compromise cell viability, making it essential to tightly regulate expression using strictly inducible promoters. It is also advisable to use curable plasmids, such as those with temperature-sensitive origins of replication, to remove the system once editing is complete. In bacteria lacking DNA repair mechanisms, such as non-homologous end joining or homology-directed repair, auxiliary integration systems (mediated by phage proteins like those from λ or Rac) may be necessary [35]. In certain bacterial genera, such as *Streptomyces*, codon optimization may also be necessary to achieve efficient Cas protein expression [151].

Nevertheless, one of the main limitations of this tool, as with multiplex editing platforms, is the occurrence of off-target mutations. Vento and collaborators (2020) point out that in the case of Cas9, this phenomenon arises because the enzyme can recognize and cut unintended sites in the genome, leading to adverse effects. These events largely depend on the sgRNA, since Cas9 has been shown to tolerate up to three mismatches between the guide sequence and the genomic DNA. In this regard, their work describes different approaches to analyse this effect, including *in silico* predictions using bioinformatic tools (i.e. use of artificial intelligence models; see [152]), experimental methods both *in vitro* and in cell cultures, as well as *in vivo* detection. They also propose various strategies to improve the specificity of the CRISPR/Cas9 system, such as protein engineering applied to Cas itself, the use of nickases, Cas9 homologue variants with rare protospacer adjacent motif (PAM) sequences, optimization of sgRNA design and improvement of Cas9-sgRNA complex delivery methods. For a more detailed analysis, their work is recommended [145].

## Conclusion and perspectives

Bacterial genomic engineering has achieved remarkable progress, offering increasingly precise, rapid and versatile tools to complement ME in the generation of industrially valuable microbial platforms. These advances have enabled the removal of unnecessary genetic components, the controlled addition of biosynthetic modules and the dynamic reprogramming of cellular regulation. However, despite this progress, several critical challenges remain, and addressing them will be decisive for consolidating the field. Among the most representative technical limitations are (I) the restricted efficiency and versatility of these tools in non-model bacteria, particularly those with complex or poorly characterized genomes; (II) the limited availability of standardized genetic component libraries for diverse species, which hinders reproducibility and scalability in engineering efforts; and (III) the challenge of maintaining long-term stability of modified biosynthetic fluxes, since epistatic interactions between introduced and endogenous genes often compromise cellular homeostasis and sustained productivity. These challenges highlight that, although tools are becoming increasingly sophisticated, their universal applicability requires coordinated efforts.

Progress towards the development of more efficient tools will be driven by the integration of artificial intelligence (AI)-based models. Predictive approaches, such as genome-scale metabolic models [153], flux balance analysis (FBA) and other AI-based algorithms, can guide the identification of metabolic bottlenecks and optimizing flux distributions. For example, the use of FBA has been highlighted in comprehensive systems analyses for the rational selection of live biotherapeutic products [154]. These computational approaches, combined with the optimization of genome editing processes, have the potential to reduce trial-and-error experimentation and accelerate the development of industrial strains.

Another promising complementary strategy involves bacterial genome reduction to create optimized cellular platforms. By eliminating non-essential or competing functions, reduced-genome strains can allocate more resources to engineered pathways, thereby improving both productivity and genetic stability. For example, improved growth, including enhanced genomic stability, was reported after the deletion of gene clusters in *Streptomyces chattanoogensis*. In *E. coli*, the removal of error-prone DNA polymerases resulted in a 50% decrease in the spontaneous mutation rate, leading to greater genetic stability. In *L. lactis* N8, a 6.9% genome reduction enabled a shorter generation time (17% less) [155]. Likewise, the design of minimal synthetic genomes equipped with modular and orthogonal regulatory networks can also mitigate interference between endogenous regulation and engineered pathways, providing a more predictable chassis for biofabrication; although this strategy is currently limited to a very small group of model bacteria.

Thus, as genomic editing tools continue to advance, bioethical and biosafety considerations have been and will remain fundamental. Chaudhari and Ranjan note that genomic editing in micro-organisms can lead to unintended consequences, such as ecological disturbances or off-target gene alterations. This potential for unintended effects raises biosafety concerns regarding microbial communities and ecosystems. From an ethical perspective, the dual-use nature of these tools also presents dilemmas, as they can be applied for beneficial purposes but may also be misused for harmful applications, such as the development of biological weapons, highlighting the need for ethical guidelines and responsible oversight [156]. Consequently, all research involving bacterial genomic editing must adhere to regulatory initiatives, such as those established in the Cartagena Protocol or Nagoya [157,158].

Overall, bacterial genomic engineering is in a stage of continuous improvement. For its implementation in a broader range of bacteria, it will be necessary to address unresolved technical bottlenecks, leverage advanced computational design, implement genome reduction strategies and consistently adhere to biosafety regulations and bioethical considerations to realize the potential of sophisticated microbial tools for biotechnology.

## Abbreviations

AI: artificial intelligence
ALE: adaptive laboratory evolution
CRISPRa: CRISPR activation
CRISPR/Cas: clustered regularly interspaced short palindromic repeat, associated with the Cas endonuclease
CRISPRi: CRISPR interference
crRNA: CRISPR RNA
dCas9: catalytically dead Cas9
DSBs: double-strand breaks
dsDNA: double-stranded DNA
EMS: ethyl methanesulfonate
FBA: flux balance analysis
FDA: US Food and Drug Administration
FRUIT: flexible recombineering using integration of thyA
GRAS: generally recognized as safe
HS1–2: homology sites 1 and 2
MAGE: multiplex automated genome engineering
ME: metabolic engineering
MMS: methyl methanesulphonate
PAM: protospacer adjacent motif
PCR: polymerase chain reaction
pHBA: *p*-hydroxybenzoic acid
pORTMAGE: portable plasmid-based platform
rDNA: recombinant DNA
RGE: rational genome engineering
SB: synthetic biology
sgRNA: single-guide RNA
SMGE: simultaneous multiplex genome engineering
ssDNA: single-stranded DNA
UV: ultraviolet
